# Antioxidant Activity of Spices and Their Impact on Human Health: A Review

**DOI:** 10.3390/antiox6030070

**Published:** 2017-09-15

**Authors:** Alexander Yashin, Yakov Yashin, Xiaoyan Xia, Boris Nemzer

**Affiliations:** 1 International Analytical Center of Zelinsky Institute of Organic Chemistry of Russian, Academy of Science, 119991 Moscow, Russia; yashinchrom@mail.ru (A.Y.); yashin@interlab.ru (Y.Y.); 2 Department of Research & Development, VDF FutureCeuticals, Inc., Momence, IL 60954, USA; Susan.Xia@futureceuticals.com; 3 Food Science and Human Nutrition, University of Illinois at Urbana-Champaign, Urbana, IL 61801, USA

**Keywords:** antioxidants, spices, herbs, flavonoids, polyphenols

## Abstract

Antioxidants are substances that prevent oxidation of other compounds or neutralize free radicals. Spices and herbs are rich sources of antioxidants. They have been used in food and beverages to enhance flavor, aroma and color. Due to their excellent antioxidant activity, spices and herbs have also been used to treat some diseases. In this review article, the chemical composition and antioxidant activity of spices and culinary herbs are presented. The content of flavonoids and total polyphenols in different spices and herbs are summarized. The applications of spices and their impacts on human health are briefly described. The extraction and analytical methods for determination of antioxidant capacity are concisely reviewed.

## 1. Introduction

Herbs and spices have been used in many different ways. Since the ancient times, spices and culinary herbs have been added to food to enhance flavor and improve their organoleptic properties. Spices and herbs have also been widely used as preservatives and medicine. 

Spices and herbs have been extensively studied in different countries because of the high antioxidant activity in certain spices and their beneficial effects on human health [1,2,3,4,5,6,7,8,9,10,11,12,13,14,15,16,17,18,19,20,21,22]. As part of our diet, spices and herbs, in addition to fruits and vegetables, could provide us with additional sources of natural antioxidants. Antioxidants from spices are a large group of bioactive compounds which consist of flavonoids, phenolic compounds, sulfur-containing compounds, tannins, alkaloids, phenolic diterpenes, and vitamins [7,8,9,11,12,13,16,21]. These compounds demonstrate different antioxidant activities. For example, flavonoids have the ability to scavenge free radicals and can form complexes with catalytic metal ions rendering them inactive. Studies have shown that spices and herbs such as rosemary, sage, and oregano are excellent sources of antioxidants with their high content of phenolic compounds.

Antioxidants can protect lipids and oils in food against oxidative degradation. When added to food, antioxidants control rancidity development, retard the formation of toxic oxidation products, maintain nutritional quality, and extend the shelf-life of products. Because of safety concerns, synthetic antioxidants are limited to be used as food preservatives. Natural antioxidants obtained from edible materials such as spices and herbs, have been of increasing interest.

Natural antioxidants contained in spices help to reduce oxidative stress. Oxidative stress, which is caused by high concentration of free radicals in cells and tissues, can be induced by various negative factors, such as gamma, UV, and X-ray radiation, psycho-emotional stress, polluted food, adverse environmental conditions, intensive physical exertion, smoking, alcoholism, and drug addiction. Chronic oxidative stress has been reported to lead to a variety of diseases, including cancer, heart related diseases, and the acceleration of aging. Several secondary products of lipid oxidation, such as malondialdehyde and 4-hydroxynonenal, can react with biological components such as proteins, amino acids, and DNA. Malondialdehyde has been shown to be formed both enzymatically and non-enzymatically, and has been implicated in health problems such as mutagenesis and carcinogenesis. Spices and culinary herbs are rich in antioxidants. Therefore, spices could potentially be used as ameliorative or preventive agents for some health issues [5,6,10,13,14,16]. 

To have a better understanding of the antioxidant activity from spices, we present the bioactive compounds, the content of flavonoids, and the total polyphenols in different spices. The therapeutic effects of various spices for different diseases are summarized. Other applications of spices and herbs are briefly described in this review. 

## 2. Chemical Composition of Spices and Their Antioxidant Activity

The antioxidant activity of spices is related to their chemical composition; primarily to the presence of polyphenolic and other biologically active compounds. Table 1 lists primary antioxidants and the biologically active compounds found in spices and culinary herbs that include flavonoids, phenolic acids, lignans, essential oils, and alkaloids, as summarized from several publications [23,24,25]. These compounds were largely determined by chromatographic methods.

The USDA (U.S. Department of Agriculture) Database contains information of flavonoid contents in spices and culinary herbs as determined by HPLC-UV (High performance liquid chromatography-UV detector) and HPLC-MS (High performance liquid chromatography-mass spectrometry) detection. The highest amounts of flavonoids have been found in parsley, oregano, celery, saffron, dill, fennel, and Tasmanian pepper (Table 2). Consumption of these spices and culinary herbs may contribute a significant portion of the plant antioxidants found in the human diet.

Ten flavonoids with significant quantity have been identified in different spices. The most frequently found compounds were quercetin, luteolin, and kaempferol. The highest flavonoid contents were as follows: apigenin in dried parsley, luteolin in Mexican oregano, luteolin in celery seeds, and cyanidin in Tasmanian pepper. The greatest amount of kaempferol and quercetin was found in capers.

Table 3 shows the most biologically active compounds contained in various spices and their chemical structures. Many of them have been reported to be anti-carcinogenic and their actions have been studied separately. These compounds also have other important therapeutic effects and antioxidant activities. Various research and review articles related to the antioxidant activity of spices have been published during the last decade [12,13,16,17,18,19,20,21,27,28,29,30,31,32,33,34,35,36,37,38,39,40,41,42,43,44,45,46,47,48,49,50,51].

### 2.1. Rosmarinic Acid in Spices

Among these biologically active compounds found in spices, rosmarinic acid was the dominant phenolic compound in the six spices of the family Labiatae which contributed significantly to the antioxidant capacity of these spices. Shan et al. [27] investigated the total equivalent antioxidant capacity and phenolic content of 26 common spice extracts from 12 botanical families. In their study, major phenolic compounds were identified and quantified in different spices. Rosmarinic acid was found in mint, sweet basil, oregano, rosemary, sage, and thyme. The spices with the highest content of rosmarinic acid were oregano (2562.7, mg per 100 g of dry weight), sage (2186.1), rosemary (1286.1), mint (1908.5), sweat basil (1086.1), and thyme (681.1) respectively. Vallverdú-Queralt et al. [52] studied the phenolic profile of widely used culinary herbs and spices, which included rosemary, thyme, oregano, cinnamon, cumin and bay. The main phenolic acid in the studied culinary herbs was found to be rosmarinic acid, which varied from 0.39 µg/g dry weight in bay, to 157 µg/g dry weight in rosemary, being the dominant phenolic compound in oregano, thyme and rosemary. The concentrations of rosmarinic acid in rosemary, oregano, cumin, cinnamon, thyme and bay are: 156.90 ± 3.20, 52.02 ± 1.74, 3.29 ± 0.12, 0.73 ± 0.09, 84.04 ± 2.75 and 0.39 ± 0.01 µg/g. 

Nagy et al. [53] analyzed the phenolic components in dried spices and found that rosmarinic acid was one of the main constituents in the methanolic extracts of oregano, sage and thyme, which was consistent with other research. 

Zheng and Wang [54] investigated antioxidant activity and phenolic compounds in selected herbs. Rosmarinic acid and hydroxycinnamic acid compounds have been demonstrated to possess strong antioxidant activity. Rosmarinic acid was the most abundant phenolic constituents in the sage extracts 117.8 mg/100 g of fresh weight. Oregano extracts also had high contents of rosmarinic acid (124.8–154.6 mg/100 g of fresh weight). Thyme and rosemary were known to have high antioxidant capacities with high contents of rosmarinic acid (91.8 mg/100 g of fresh weight). It was found that certain species of oregano had extremely high total phenolic contents and oxygen radical absorption capacity (ORAC) values as well. 

### 2.2. Flavonoids in Spices

Typically flavonoids and phenolic acids are the main phenolics in spices that possess antioxidant activity. Flavonoids generally occur as glycosylated derivatives with apigenin and luteolin are commonly found in aromatic herbs such as parsley, rosemary, thyme; quercetin and kaempferol in onions. 

The antioxidant activity of phenolic compounds is mainly due to their redox propertiessuch as adsorbing and neutralizing free radicals, quenching singlet and triplet oxygen, or decomposing peroxides. In general, flavonoids have higher antioxidant activities against peroxyl radicals than do phenolic acids due to multiple hydroxyl groups. 

Research [55] showed that quercetin was the major dietary flavonoid, followed by kaempferol, luteolin, and apigenin in the Netherlands. The greatest dietary source of flavonoids include onions which is 29% of total intake (approximately 23 mg/day expressed as aglycones). 

Wojdylo et al. [56] measured antioxidant activity and phenolic compounds in 32 selected herbs. It was found that quercetin, luteolin, apigenin were predominant flavonoids in those herbs in addition to phenolic acids.

Yao et al. [57] studied the flavonoids in food sources for humans and the health aspects of flavonoids. It was indicated that cumin and peppermint were good sources of flavanones (Naringenin, eriodictyol); parsley and thyme are sources of flavones (apigenin, chrysin, luteolin, diosmetin), and onions are sources of flavonols (isorhamnetin, kaempferol, quercetin, myricetin, rutin). 

Dimitrios [58] introduced extensive sources of natural antioxidants from fruits, vegetable, seeds, wine, and tea. It was demonstrated that different spices and herbs are important sources of natural phenolic antioxidants: flavones in parsley; carnosic acid, carnosol, rosmarinic acid, rosmanol in rosemary; carnosol, carnosic acid, lateolin, rosmanul, rosmarinic acid in sage; rosmarinic acid, phenolic acids, flavonoids in oregano; thymol, carvacrol, flavonoids, lubeolin in thyme; rosmarinic, carnosol, carvacrol, flavonoids in summer savory and Gingerol and related compounds in Ginger. 

Study [54] revealed that Greek mountain oregano, hard sweet marjoram, Mexican oregano and sweet bay had higher total phenolic content in various herbal extracts studied, with total phenolic content 11.8 ± 0.60, 11.65 ± 0.29, 17.51 ± 0.22, and 4.02 ± 0.90 mg GAE/g respectively (Results were expressed as milligrams of gallic acid equivalent (GAE) per gram of fresh weight). 

Kähkönen et al. [59] measured the antioxidant activity of different plant extracts spectrometrically according to the Folin–Ciocalteu procedure and found the total phenolic content of bog-rosemary as calculated as GAE was even higher than that of all the vegetables and some fruits studied at the same time. 

In a mini review, Embuscado summarized total phenolic content of spices and herbs from different researches [60]: allspice 421.5 µmol gallic acid eq. per gram; basil 122.0 mg per gram; black pepper 3.83 mg per gram; coriander 18.5 µmol gallic acid eq. per gram; cinnamon 157.18 mg per gram; clove 113.19 mg per gram; cumin 49.5 µmol Trolox eq. per gram; fennel 46.1 µmol gallic acid eq. per gram; ginger 3.17 mg per gram; parsley 15.5 mg per gram; thyme 23.24 mg per gram; turmeric 21.17 mg per gram.

Shan et al. [26] found that cloves have the highest amount of flavonoids (366.5 as mg per 100 g), followed by dill (241.2), caraway (171.9), coriander (167.2), oregano (51.3), rosemary (37.8), mint (23.2), basil (21.0), and sage (20.5). 

A database [27] that contains data for 425 spices and culinary herbs from 59 countries was produced by a number of manufacturers. The antioxidant content was measured by modified ferric reducing antioxidant power (FRAP) assay. Antioxidants were extracted in water/methanol. Twenty-seven spices had the highest antioxidant content which varied from 100 to 465 mmol per 100 g. Shan et al. [26] showed the total antioxidant capacity of 26 spices from 12 botanical families as determined by ТЕАС. The phenolic content was measured using Folin–Ciocalteu assay. Colves, cinnamon and oregano were the three spices with the highest value. A high correlation (R = 0.9613) was found between the ТЕАС value and the total phenolic content. 

It is not a surprising that spices and herbs are at the top of the list of 100 products with the highest antioxidant content [61,62]. Their antioxidant activities are ten times higher than that of fruit and vegetables. The antioxidant capacities of some spices showed a positive correlation with their corresponding total polyphenol concentrations. 

## 3. Methods for Antioxidant Extraction and Determination

### 3.1. Extraction Techniques for Antioxidant in Spices

Extraction techniques aim to extract the active compounds from spices with certain selectivity and sensitivity. Several well-known methods have been used for extraction of active components from spices, such as liquid-phase extraction (extraction using solvents), solid-phase extraction, and supercritical fluid extraction (extraction using СО_2_ in its supercritical state) [63]. 

It was proven that different solvents or solvent mixture applied on the same spice sample can lead to different extraction efficiencies. A list of solvents used for extraction of spices has been provided by AllwynSundarRay et al. [25]. The solvents include methanol, methanol/water mixture (1:1), trichloroacetic acid, acetone, toluene, ethanol, ethyl acetate, and water. Methanol/water (1:1) and ethanol/water (1:1) mixtures are used most frequently. The total contents of flavonoids, polyphenols, and tannins in aqueous and methanolic extracts of cardamon, coriander, and bay leaf were different [50]. The results showed that the content of all these polyphenols in aqueous solutions of coriander was almost twice as much as that in the methanolic solutions. The opposite results were obtained for cardamon and bay leaf with polyphenol content in methanolic extracts significantly higher—2–3 times higher for cardamon and 3–10 times higher for bay leaf.

The extraction efficiency could be affected by the extraction temperature. Hot water extraction (80–100 °C) has been used for measuring antioxidant activity of 13 spices [41]. The extraction lasted 3 h. Antioxidant activity was determined by the 2,2-diphenyl-1-picrylhydrazyl (DPPH) method. However, the measurements may not be appropriate because a significant part of polyphenols could have been oxidized over 3 h at such high temperatures. Ereifej et al. [64] investigated the influence of different extractants (methanol, ethanol and acetone) at different temperatures (20, 40 and 60 °C). It was found that cloves had the highest level of total phenolics using methanol at three different temperature. When ethanol was used as extractant, cloves show the highest level of phenolics at 60 °C. When acetone was used as an extractant at 60 °C, cloves still had the highest levels of total phenolics. The total phenolics of cloves were quite close using methanol and acetone as extractants. 

To address the complexity of the extraction characteristics and optimize parameters for solvent extraction of phenolic compounds and antioxidants, response surface methodology by central composite design has been employed to statistically optimize the extraction conditions [65].

### 3.2. Analytical Methods Applied to Antioxidant Capacities Determination in Spices

A variety of methods have been used to determine antioxidant capacities in spices and herbs. The reported methods include 2,2-azino-bis(3-ehtylbenzothiazoline-6-sulfonic acid (ABTS) assay, DPPH radical scavenging activity, ORAC, ferric reducing antioxidant power (FRAP) and sensitive electrochemical and photochemiluminescent approaches, voltammetric and spectrophotometric methods [65,66,67,68,69,70]. Recently, a rapid approach based on near-infrared spectroscopy was developed for the determination of total polyphenols content and antioxidant activity in a Chinese herb [71]. Lu et al. [72] investigated the antibacterial effects of garlic concentrate by using Fourier Transform Infrared Spectroscopy. Similarly Venetsanou et al. [73] estimated antioxidant activity of different mixed herbal infusions using attenuated total reflectance Fourier transform infrared spectroscopy and chemometrics.

Antioxidant activities and antioxidant capacities of compounds from the same spices could be different depending on the analytical methods used. Antioxidant activity of ethanol and water/ethanol extracts of 13 spices have been evaluated by three different methods [51]. In many cases, water/ethanol extracts contained more antioxidants than ethanol extracts (measurements by ABTS^+^). When the same extracts were evaluated using photochemiluminescence or cyclic voltammetry, the results obtained for different spices shifted in both directions. Romero et al. [74] studied the antioxidant activities of a set of 17 active components present in different spices and condiment with different methods: (1) spectrophotometric method with DPPH radical scavenging assay and cupric reducing antioxidant capacity (CUPRAC) assay; (2) electrochemical method using either a mercury electrode or a glassy carbon electrode covered with poly-neutral rad and doped with Pt nanoparticles. The electrochemical method saves time for measurement and organic solvents for extraction. The sensitivity of electrochemical method is comparable with those shown by DPPH or CUPRAC. 

Antioxidant activities from the same spices measured by different methods could be correlated. Dragland et al. [29] studied antioxidant activity of several dozen spices, culinary and medicinal (Chinese and Japanese) herbs. They reported that the antioxidant activity of these spices differs by as much as three orders of magnitude. Antioxidant activity in their study was determined by an automated FRAP assay. To compare these data to Carlsen et al. [28] using modified version of the FRAP assay, we correlated the two data sets and found their correlation was highly positive with correlation >0.9. A comparative study of antioxidant properties of 30 plant extracts using various methods—DPPH, ABTS, FRAP, superoxide dismutase (SOD), and ORAC assays demonstrated the different correlations among these methods [45]. The total polyphenol contents of cinnamon, cloves, bay leaf, vanilla, lavender and ginger were determined by Folin–Ciocalteu assay. The strongest correlation was found between the FRAP and ABTS assays (0.946), and the weakest was between ABTS and DPPH assays (0.906).

So far no single method will truly reflect the total antioxidant capacity of a particular sample. In order to fully reflect both lipophilic and hydrophilic capacity, elucidate a full profile of antioxidant capacity, and evaluate various reactive oxygen species, a number of methods are required. However, from the routine quality control point of view, it would be more practical to employ fewer, and standardized methods.

## 4. Effect of Spices on Human Health and Other Applications

Spices have been reported to have various beneficial effects on human health which include anti-sclerotic, antithrombotic, anti-carcinogenic, anti-inflammatory, antiarrhythmic, anti-rheumatic, gastroprotective, and lipid-lowering action. In addition, spices have radioprotective (protects against radiation), anti-allergic, and antimalarial effects. Spices inhibit the oxidation of low-density lipoprotein and protein glycation [75,76,77,78,79].

Many spices are highly potent antiseptics because they have an antibacterial, antimicrobial, and even antiviral effect. A synergistic effect on oral bacteria was observed when cloves were used along with antibiotics [80,81,82,83,84].

Spices and herbs have been used as functional food [85,86,87,88,89]. Table 4 shows various therapeutic effects of spices from different literatures.

The therapeutic effects of certain spices are so significant that they have often been included in non-clinical, clinical, and therapeutic studies. A non-clinical trial of rosemary showed that rosemary could act as a cancer prevention agent. Some clinical and therapeutic trials for evaluation of spices against several diseases have been conducted. Studies show that curcumin possesses anti-inflammatory effects and therapeutic effect in gastrointestinal diseases. It is an inhibitor of low density lipoprotein oxidation and also showed effects against neurodegenerative diseases. Ginger and garlic have extensively therapeutic effects [90,91,114,115,116,117,118,119,120], especially for cardiovascular diseases. These will be reviewed in following section. 

Cloves, Nigella, black pepper, garlic, and ginger have been used against cancer [121,122,123]. Aged aqueous-alcoholic extract of garlic was also reported to be potentially effective against certain cancers [124]. Table 5 summarizes anticancer actions of some spices. The following compounds contained in spices have anti-carcinogenic properties: curcumin, apigenin, luteolin, quercetin, thymoquinone, and isothiocyanate. 

Some spices play a critical role in the management of heart disease (Table 6) because these spices have been shown to inhibit enzymes involved in lipid synthesis, decrease platelet aggregation, prevent lipid peroxidation, reduce LDL (low-density lipoprotein) levels and increase coronary blood flow. 

Spices have been used to preserve food to inhibit or delay lipid oxidation and rancidity in foods. Since ancient times it has been known that spices help preserve many foods. Now the application of spices have become even more broad: extension of cheese shelf-life by adding cinnamon; preservation of vitamin E in sunflower oil by adding different spices; inhibition of omega-3 fatty acid oxidation in vegetable oils by oregano and rosemary as well as sterol oxidation in extra virgin olive oil; extension of meat shelf-life by various spices [145,146,147,148,149].

Due to high antioxidant activity, spices suppress harmful effects of carcinogenic pollutants that may be present in foods and beverages, especially aflatoxins, heterocyclic amines, acrylamide, 1,2-Dimethylhydrazine and cadmium [150,151,152,153,154,155]. Spices can also neutralize the harmful effects of hazardous solvents and motor exhaust emissions from road transport in urban areas [156]. Therefore, it is important and reasonable to encourage people to consume spices regularly in order to protect them from harmful environmental impacts, especially in large, polluted cities.

## 5. Conclusions

Based on the reviewed literature, we know that spices not only enhance the flavor, aroma, and color of food and beverages, but they can also protect people from acute and chronic diseases, due to their high antioxidant activity. This review presents abundant data on the antioxidant activities of spices and culinary herbs, as well as information related to their content of flavonoids and total polyphenols. Many of the antioxidants contained in spices have significantly high biological activities and are actively used in preclinical, clinical, and therapeutic trials investigating new treatments of diseases. It is possible that new spice-based drugs may be developed. This review also presents a strong body of evidence that spice consumption can reduce or even eliminate the harmful effects on humans from contaminants in foods and from the environment; this is even more important for people living in polluted cities. All of this information will hopefully add to an already high level of interest toward spices and culinary herbs. Spices and herbs should certainly be incorporated as integral parts of healthy, nutritious eating, and as functional food ingredients.

## Figures and Tables

**Table 1:** Chemical composition of spices (seasonings) and culinary herbs [23,24,25].

Spices and Herbs	Important Chemical Constituents
Cloves	Eugenol, isoeugenol, acetyleugenol, sesquiterpene, pinene, vanillin, gallic acid, flavonoids, phenolic acids
Cinnamon	Eugenol, limonene, terpineol, catechins, proanthocyanidins, tannins, linalool, safrole, pinene, methyleugenol, benzaldehyde
Cardamon	Limonene, 1,8-cineole, terpinolene, myrcene, caffeic acid, quercetin, kaempferol, luteolin, pelargonidin
Coriander	Linalool, borneol, geraniol, terpineol, cumene, pinene, terpinene, quercetin, kaempferol, caffeic, ferulic, n-coumaric and vanillic acids, rutin, tocopherols, pyrogallol
Saffron	Crocins (water soluble carotenoids), safranal, flavonoids, gallic, caffeic, ferulic, *n*-catechuic, syringic, salicylic, and vanillic acids
Turmeric	Curcumins, essential oils, eugenol, carotene, ascorbic acid, caffeic, *p*-coumaric, protocatechuic, syringic, vanillic acid
Ginger	Gingerol, turmeric, paradol, geraniol, geranial, borneol, linalool, camphene, zingerol, zingiberon
Anise	Camphene, pinene, linalool, *trans*-, *cis*-anetholes, eugenol, acetanisole, rutin, luteolin-7-glucoside, apigenin-7-glucoside, isoorientin
Caraway	Monoterpenes, sesquiterpene, aromatic aldehydes, terpene esters, terpenol, terpenal, terpenon, limonene, safranal, kaempferol, quercetin, tannins, caffeic, ferulic, *p*-coumaric, and chlorogenic acids
Fenugreek	Sesquiterpenes, aromatic aldehydes, terpenes
Black pepper	Piperine, pinene, camphene, limonene, terpenes, piperidine, isoquercetin, sarmentine
Oregano	Apigenin, quercetin, luteolin, myricetin, diosmetin, eriodictyol, carvacrol, thymol, rosmarinic, caffeic, *p*-coumaric, protocatechuic acid
Basil	Apigenin, catechins, quercetin, rutin, kaempferol, anthocyanins, eugenol, limonene, terpinene, carvacrol, geraniol, menthol, safrole, tannins, ursolic, *p*-coumaric, rosmarinic acids
Bay leaf	1,8-cineole, cinnamtannin
Dill	Quercetin, kaempferol, myricetin, catechins, isorhamnetin, carvone, limonene
Garlic	Allicin, diallyl sulfide, diallyl disulfide, diallyl trisulfide, allyl isothiocyanate, *S*-allyl cysteine
Horseradish	Phenyl methyl isothiocyanate, allyl isothiocyanate, sinigrin, asparagine
Allspice	Eugenol, gallic acid, pimentol, quercetin
Marjoram	Limonene, pinene, terpinene, *p*-cumene, apigenin, ferulic, sinapinic, caffeic, syringic, rosmarinic, 4-hydroxybenzoic, vanillic acids
Mustard	Allyl isothiocyanate, carotene, isorhamnetin, isorhamnetin-7-O-glucoside, kaempferol glucoside
Fennelflower	Pinene, *p*-cumene, thymoquinone, thymohydroquinone, thymol, carvacrol, nigellicine, nigellidine, hederin
Onion	Quercetin, apigenin, dipyridyl disulfide, rutin, quercetin-4-glucoside
Parsley	Apigenin, luteolin, kaempferol, myricetin, quercetin, caffeic acid
Red pepper	Capsaicin, tocopherol, lutein, carotene, capsanthin, quercetin, ascorbic acid
Peppermint	Menthol, menthone, limonene, isomenthone, eriocitrin, hesperidin, apigenin, luteolin, rutin, carotenes, tocopherols, caffeic, rosmarinic, chlorogenic acid
Rosemary	Carnosol, rosmanol, geraniol, pinene, limonene, apigenin, naringin, luteolin, rosmarinic, vanillic, ursolic, caffeic acids
Sage	Geraniol, pinene, limonene, carnosol, saponin, catechins, apigenin, luteolin, rosmarinic, carnosine, vanillic, caffeic acids
Nutmeg	Catechins, lignans, myricetin, orgentin, caffeic acid
Myrtle	Anthocyanins, pinene, limonene, gallic and ellagic acids, myrtocommulone, myricetin-3-O-galactoside, myricetin-3-O-rhamnoside
Lavender	Limonene, quercetin, apigenin, kaempferol glucoside, ferulic, rosmarinic, caffeic, *p*-coumaric acid

**Table 2:** Flavonoid content in spices from the USDA (U.S. Department of Agriculture) database on flavonoids content of selected foods, release 3.1 (2014) [26].

Name	Flavonoid Content (mg per 100 g)	Total Flavonoid Content (mg per 100 g)
Parsley	Apigenin 4503.5, isorhamnetin 331.2, luteolin 19.7	4854.5
Mexican oregano	Luteolin 1028.7, naringenin 372.0, eriodictyol 85.3, quercetin 42.0, apigenin 17.7	1550.79
Celery seeds	Luteolin 762.4, apigenin 78.65	841.05
Capers	Kaempferol 259.19, quercetin 233.84	493.03
Saffron	Kaempferol 205.48	205.48
Dill	Quercetin 55.15, isorhamnetin 43.50, kaempferol 13.33, myricetin 0.70	112.68
Thyme	Luteolin 45.25, apigenin 2.50	47.75
Fennel	Quercetin 48.80, myricetin 19.80, isorhamnetin 9.30, kaempferol 6.50, luteolin 0.10	84.50
Coriander, leaves	Quercetin 52.90	52.90
Wormwood	Quercetin 10.0, kaempferol 11.0, isorhamnetin 5.0, luteolin 1.0	27.0
Rosemary	Naringenin 24.86, luteolin 2.0, apigenin 0.55	27.41
Ginger	Kaempferol 33.60	33.60
Mustard	Kaempferol 38.20, quercetin 8.80, isorhamnetin 16.20	62.90
Sage	Luteolin 16.70, apigenin 1.20	17.90
Red onion	Quercetin 20.30, isorhamnetin 4.58, delphinidin 4.28, cyanidin 3.19, peonidin 2.07, kaempferol 0.65, apigenin 0.24	35.31
Chile pepper	Quercetin 14.70	14.70
Yellow pepper	Quercetin 50.63, luteolin 6.93	57.56
Tasmanian pepper	Cyanidin 752.68	752.68
Garlic	Quercetin 1.74, myricetin 1.61, kaempferol 0.26	3.61

**Table 3:** The major biologically active compounds found in spices and herbs.

Spices	Active Substance	Formula
Ginger	Gingerol	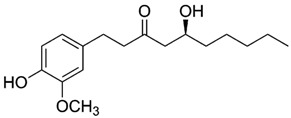
Rosemary	Rosmarinic acid	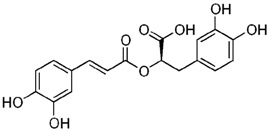
Onion	Quercetin	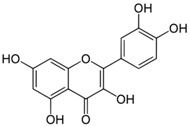
Turmeric	Curcumin	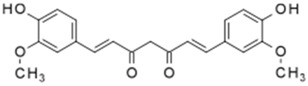
Cloves	Eugenol	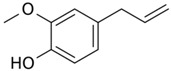
Fennelflower	Thymoquinone	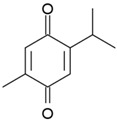
Black pepper	Piperine	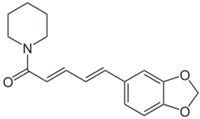
Garlic	Allicin, S-allyl cysteine	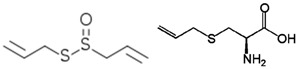
Red pepper	Capsaicin	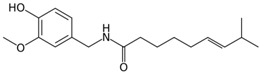
Parsley	Apigenin	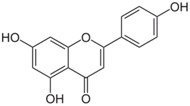
Oregano, celery (seeds)	Luteolin	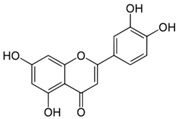
Capers	Kaempferol	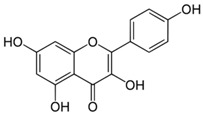
Tasmanian pepper	Cyanidin	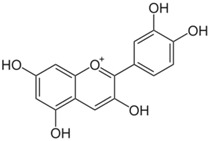
Saffron	Crocetin, crocin	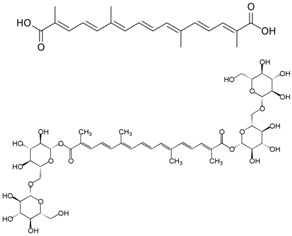

**Table 4:** Reported therapeutic effects of spices in different diseases.

Diseases	Spices	References
Cardiovascular diseases, including heart attack	Garlic, turmeric, ginger	[90,91,92]
neurodegenerative diseases	Mint, onion	[93]
Antidiabetic action	Cinnamon, bay leaf, wormwood, fenugreek, mustard, pomegranate	[94,95]
Gastrointestinal diseases	Black pepper, bay leaf	[96,97]
Hypertension	Cardamon, cinnamon	[98,99]
Hepatic diseases	Caraway, cardamon	[43,100]
Endocrine diseases	Ginger, turmeric	[87]
Against DNA oxidation	Basil	[101]
Obesity	Saffron, turmeric	[102]
Bone diseases	Cloves	[103]
Protection against oxidative damage to red blood cells	Fenugreek, garlic	[104]
Immunomodulatory action	Turmeric	[105,106]
Renal diseases	Garlic, fennelflower, ginger	[107,108]
Antiulcer action	Ginger	[109]
Pigment cell growth inhibition	Turmeric	[110]
Reduction of cortisol level in saliva	Lavender, rosemary	[111]
Against alcohol abuse	Thyme, ginger	[112]
Against gum disease	Licorice	[113]

**Table 5:** Spices against cancer.

Spices	Cancer Type	References
Turmeric	Rectal cancer, oral cancer, leukemia, carcinoma of the head and neck	[125,126]
Saffron	Skin carcinoma, rectal cancer, hepatic carcinoma	[127,128,129]
Garlic	Prostate cancer, colon cancer	[130,131]
Onion	Gastric carcinoma	[132]
Turmeric	Leukemia	[133]
Mustard	Rectal carcinoma, bladder cancer	[134,135]
Bay leaf	Inhibits melanoma cell growth	[136]
Mustard (seeds)	Rectal carcinoma, bladder cancer	[137]

**Table 6:** Major bioactive compounds of spices with potential beneficial effects for the management of CVD (Cardiovascular disease).

Spices	Major Bioactive Compounds	Potential Beneficial Effects	Potential Mechanism for Anti-CVD Characteristics	References
Cinnamon	Procyanidin	Antioxidant	Increase coronary blood flow	[85,99]
	Cinnamaldehyde	Antimicrobial potential	Provoke pituitrin induced reduction of blood flow	[138,139,140]
			Reduce peripheral vascular resistance	
			Increase cardiac contractile force	
Ginger	Gingerol	Antioxidant	Reduction in platelet aggregation	[19,140,141]
	Shogaol	Anti-inflammatory	Reduce LDL cholesterol levels	
	Zerumbone		Reduce LDL atherogenic modifications	
			Reduction in the oxidative response of macrophages	
Garlic	Allicin	Antioxidant	Inhibit enzymes involved in lipid synthesis	[140,141,142]
			Decrease platelet aggregation	
			Prevent lipid peroxidation of oxidized erythrocytes	
			Increase antioxidant status	
			Inhibit angiotensin-converting enzyme	
Turmeric	Curcumin	Antioxidant	Reduce platelet aggregation	[140]
	Capsaicin	Anti-inflammatory	Decrease triglycerides	
			Reduce thromboxane	
			Decrease serum cholesterol	
			Decrease cardiomyocytic apoptosis	
Onion	Polyphenols	Antioxidant	Reduce platelet aggregation	[143]
	Flavonoids		Reduce cholestrol level	
	Flavonols		Enhance blood fibrinolytic activity	
Red pepper	Curcumin	Antioxidant	Hypotriglyceridemic	[22]
	Capsaicin	Anti-inflammatory	Reduce cholestrol in blood and liver	
Fenugreek	Rhaponticin	Antioxidant	Lymphatic cleansing	[141,142,143,144]
	Isovitexin	Anticarcinogenic	Decrease blood pressure	
			Hypoglycermic effect	
